# On Relations Which Dental Caries—as Discovered Amongst the Ancient Inhabitants, &c.—may Be Supposed to Hold to Their Food and Social Condition

**Published:** 1883-01

**Authors:** John R. Mummery

**Affiliations:** L.D.S., F.L.S.


### THE DENTAL REGISTER.

Vol. XXXVII.] JANUARY, 1883. [No. 1.

### COMMUNICATIONS.

### On the Relations which Dental Caries—as Discovered Amongst the Ancient Inhabitants of Britain, and Amongst Existing Aboriginal Races—May be Supposed to Hold to Their Food and Social Condition.

BY JOHN R. MUMMERY, ESQ., L.D.S., F.L.S.

Mr. President and Gentlemen: In my concluding paper, "On the Relations of the Human Teeth to those of the Lower Animals," read in May, 1860, I advanced the opinion, that dental caries rarely if ever occurred among the ancient races who once inhabited our island.

That opinion was founded on the state of skulls I had myself seen, from the Kentish barrows, and was strengthened by the testimony of a lamented scientific friend, then in declining health, whom I had requested to examine the skulls in the Hunterian Museum, and who from a cursory view of these skulls had come to the same conclusion. A more extensive view of the subject, however, led me afterwards to doubt the accuracy of the judgment I had then formed, and I have during the past nine years, availed myself of such opportunities as have occurred, upon an occasional holiday, to investigate the whole subject on as wide a scale as practicable; feeling it to be one of the deepest interest, and possibly also of practical value—whilst it always furnished a most interesting object of pursuit upon a journey; and I now purpose laying before you the result of those more extended observations.

Topographically my investigations have been bounded on the north by the line of the great Roman wall of Hadrian, which extends from the Solway to the Tyne, for a length of nearly seventy miles (along the course of which I have explored the remains of many Roman stations and towns with feelings of the deepest interest); on the west by the borders of Wales, and on the south by a line drawn from Bristol to Lymington in Hampshire.

In these endeavors to trace the physical characteristics, food, and habits of the various races who have successively peopled England—with their relations to the pathological conditions of the teeth, I examined and tabulated the state of the teeth in every available skull in the Hunterain Museum. Feeling convinced, however, that only the widest possible survey of the whole subject could furnish data that should be received as trustworthy evidence, I visited and examined several other great collections of skulls. Among these I may mention the museum of the late Mr. Bateman of Lomberdale, Derbyshire, celebrated for his ten years' successful diggings in British barrows; and that of the Rev. Canon Greenwell of Durham, who has also afforded me every facility for examining the results of his enterprising labors in the same department of archæology. Professor Rolleston, also, has most kindly assisted me in examining the fine series of skulls in the Oxford University Museum, and has supplied me with invaluable light on the subject of Ancient English Ethnology.

I have experienced similar kindness from the joint authors of the justly celebrated work "Crania Britannica." From the very extensive collection of Dr. Davis, of Hanley, Staffordshire, I selected a large number of skulls of certain existing pure races for comparison, receiving from him much useful information. His coadjutor, Dr. Thurnam, of Devizes, has also most efficiently aided me in the prosecution of my object, and I was enabled to examine every skull in his possession—a large proportion of which were exhumed by himself from the tumuli in the neighborhood of that ancient memorial of Druidical rites—Stonehenge; Dr. Thurnam supplying me with a very clear explanation of the relative antiquity of the successive races inhabiting England.

Some differences of opinion exist among our most learned ethnologists with regard to the original stock of these ancient races, but it would be out of place on my part, and beyond the fair scope of this paper, to enter more fully into the question than is absolutely necessary. The form and capacity of the cranium and the facial bones have received minute attention from many able observers; but the exact condition of the teeth, with regard to injury or disease, deficiency or redundance, has been seldom recorded, while excessive abrasion of the teeth has been mistaken by some observers for caries; or (as in the case of the eminent Blumenbach) for malformation. Describing the front teeth of Egyptian mummies, for example, he says, "The teeth are different from those of any other people; the crowns of the front teeth are not wedge-shaped, terminating in a thin edge, but are thick, cylindrical, or obtusely conical." This condition I have myself observed in many Egyptian skulls; recognizing it, however, as undoubtedly the result of severe attrition. I may here remark that in recording the amount of wearing down of the teeth, I have estimated the probable age of the subject by the state of the cranial sutures; that in each race I have taken the maximum and minimum diameter of the dental arch at the first molar in each group, and that I have distinguished the cases in which disease occurred at the approximal surfaces of the teeth, from that found on the buccal, lingual, or grinding surfaces. With a view to comparison with these ancient races, I have selected certain existing tribes concerning whom we have accurate information; and that no available source of illustration might be neglected, I have examined the fine series of skulls in the respective museums of the Royal Military Hospital, at Netley; the Naval Hospital, Haslar; the principal London hospitals; and the "Dreadnought" (although in many instances the skulls were unsuitable for my purpose). I have also seen several local museums and private collections, and having, on the whole, examined nearly 3,000 skulls, I have tabulated 1,658 of them, rejecting those of which the authenticity was doubtful, or from which too many teeth had fallen out, to enable me to form a correct judgment concerning them.

The ancient British tumuli of Wiltshire are so much more numerous than those in any other part of England, and they have been so thoroughly explored and clearly explained by Dr. Thurnam, that I shall select this locality for illustrating the condition of the early inhabitants of our island.

These tumuli are of two distinct forms, long and round, and represent two distinct races; although occasionally much misapprehension has been caused by a practice of the later race, who often buried their dead in the older tumuli. The earliest in date are the long-shaped barrows, which are either made of solid earth or rubble, or contain rudely-constructed chambers formed of large stones. At this early period, the use of metals was unknown in our island—arrow heads, and other implements of flint, greenstone, and bone having alone been found in these mounds. The burning of the dead was sometimes practiced, but if simply buried, the body was laid in a contracted position on its side, with the knees drawn up.

The skulls from these barrows are of the long or dolichocephalic type, and are narrower than those of any modern European people. This nation of the stone period had been driven back into the western parts of Britain, before the invasion of Cæsar, by the advancing tribes of Belgic Celts, to whom I shall presently refer. They were a pastoral, and not an agricultural people; they possessed herds of the small short-horned oxen (*Bos longifrons*), and were to a great extent supported by the chase of the red-deer, roebuck, and wild boar, at a time when vast districts of the country were covered with forests; the extensive forest of "Anderida," for example, having reached from the coast of Kent and Sussex into Somersetshire. Their customs appear to have been extremely barbarous, and to have closely resembled those of the Ashantees, Australians, and New Zealanders of our own day. It was with feelings of peculiar interest that I examined, in Dr. Thurnam's collection, the cleft skulls and half-charred bones of victims who had been sacrificed at the funeral of a chief. Several examples of this kind have been found on the Downs, in the district around Stonehenge; and as the cylindrical bones among them are often found split open, as though to obtain the marrow, there is but too much reason to believe that these aborigines, like the people of the countries I have referred to, were guilty of the revolting practice of cannibalism.

On comparing the condition of the teeth in these southern people, with those of apparently the same race inhabiting the more northern districts, a striking difference is observable. Among sixty-eight Wiltshire skulls, I have found but one case of decay on the approximal surface, and one on the grinding surface; only eleven cases of extreme wearing down of the teeth, and those occurring in persons of advancing age; and I discovered not a single instance of irregular position of the teeth. The distinctions of priority of race are less clearly marked in the skulls from the late Mr. Bateman's collection, and I have therefore grouped them together with some obtained from three other sources. In forty-four skulls in this group, I have found five cases of approximal caries, with four of the grinding surfaces; but the teeth are much more worn down (sometimes even to the margin of the enamel) than in the Wiltshire skulls, and yet the traces of alveolar abscess are of rare occurrence.

The round barrows of Wiltshire were constructed by a brachycephalic, short, or round headed people; and it is worthy of remark, that whereas the skulls from the primitive long barrows are narrower than those of any modern European people, those from the round barrows are much broader than the skulls of the existing population of any part of England or Wales. The interments of the later or round-headed race consist of burnt remains in the proportion of three to one of the unburnt bodies; but the objects found in both classes of interment are similar, and, in addition to the implements of stone, etc., already alluded to, the tumuli of the round-headed race often comprise implements of bronze and highly ornamental pottery.

There appears good reason for assuming that these round-headed people were a more powerful and more civilized race, and are to be identified with the agricultural population of the maritime districts of Britain, said by Cæsar in the fifth book of his Commentaries to have exactly resembled the people inhabiting Belgic Gaul, and who are described by Tacitus, Strabo, and other classical writers, as a people of tall stature and vigorous frame. The change of diet and habits accompanying a higher degree of civilization appears to have had a deteriorating effect on their teeth; for among thirty-two skulls in Dr. Thurnam's collection, I find six cases of approximal caries, one of caries of the grinding suface, and two cases of irregularity from contracted arch, besides ten in which the crowns are deeply abraded. There is good reason also to believe that as the native wild animals became more scarce, and agriculture was extended, the people of the race now under consideration were in the habit of consuming more vegetable food, probably in the form of coarsely prepared barley or oats.

A remarkable difference is observable in the teeth of the skulls from the Yorkshire barrows, so diligently explored by Canon Greenwell, as compared with those of the skulls found in the south of England. We noticed how few signs of dental caries the skulls of the ancient Britons presented in the case of the Wiltshire skulls; but in about sixty Yorkshire skulls, consisting principally of the earlier or long-headed race, I found no less than twenty-six exhibiting more or less disease. Their teeth are also much worn, and traces of abscess are of frequent occurrence. It is somewhat difficult to account for this. Possibly, as in the case first referred to, it may be caused by a greater scarcity of nourishing animal food, and the use of a harder vegetable diet in that district; but it is a curious fact that, while the proportion of disease which in Roman skulls in other parts of England is in the ratio of thirty-two per cent., those found in Yorkshire exhibited decay in eighteen cases out of twenty-three.

With the advent of the Roman conquerors a great change took place in the condition of the British people, and advancing civilization was accompanied by habits of luxury and effeminacy which greatly deteriorated their moral principles and physical vigor. The Epicurean indulgence of the Romans was notorious, whilst every country in the empire was laid under contribution for its delicacies, and their gross expedients for stimulating the jaded appetite do not require more explicit reference. This part of the subject is full of interest, but I must not be tempted to enlarge upon it. It will suffice for our inquiries to recognize the fact, that the Romano-Britons adopted the habit of their new masters.

In the earlier days of the Republic the Romans both buried and burnt their dead, but the practice of incremation gradually increased, as Rome became more and more engaged in foreign wars, until in the days of the early Empire it had become almost universal. When, by the firm establishment of their Empire, the Romans were freed from anxiety as to posthumous outrage at the hands of half-subdued peoples, they began to discontinue this custom, and with the prevalence of Christianity it ceased altogether. Owing to this practice the Roman skulls found in this country are less abundant than might have been anticipated; but I have succeeded in examining altogether 143 Roman skulls, distributed through nine collections. Of these forty-one have carious teeth, twenty-nine of which are affected on their approximal surfaces. In the whole number of 143 only six occurred in which the teeth were deeply worn down, but the amount of caries in some individuals was very extensive. In the case of a woman of about thirty years of age, every molar and bicuspid tooth was diseased, and a similar condition existed in six other cases. Traces of extensive abscesses of the alveoli are frequent, involving in one case the three lower molars in one large cavity on the right side of the lower jaw. I found also three cases of contracted arch, and irregularity in incisors and canines; two cases of approximal caries in front teeth; and eight cases in which two or more of the third molars were not erupted in middle life. In the museum at York I observed a remarkable instance of the durability of the enamel under certain conditions. The body of a Roman lady had been placed in a coffin and covered with newly-slackened lime, which soon hardened, and the mass being now shown in a reversed position, a distinct impression of the body is seen. Although few of the bones remain, and the roots of the teeth have also disappeared, the whole of the crowns are preserved and in a sound state, the enamel still being beautifully white.

In connection with this part of our subject, I felt great curiosity to know how far it was possible to find any traces among these

Roman remains of the use of artificial teeth, or of the practice of dental surgery. Many of the ingredients of the old Roman tooth-powders, as enumerated by Pliny, are found in modern recipes. Burnt hartshorn, egg-shells, and oyster-shells, nitre, and myrrh are commended for cleansing and whitening the teeth, strengthening the gums, and preventing toothache; but Pliny says if pumice-stone be added, "Utilissima fiat ex his dentifricia." A destructive usefulness, truly! That the front teeth at least were sometimes replaced we know through the satires of Martial and Juvenal, the former poet even naming ivory as a material employed.* I found no traces, however, of any such handiwork in the Roman skulls I examined, although several of them were those of people of rank, as was evident from the cedar, stone, or lead coffins which inclosed their remains; nor did I find any artificial tooth, or stopping. That I might leave no source of possible information unexplored, I obtained permission, through the great courtesy of the chief authorities of the British Museum, to take out and examine the ashes of all the cinerary urns which still contain such remains, in the fine collection deposited in the "columbarium" in the basement of that institution. Here I found calcined bones and natural teeth, but neither gold wire nor plate, nor any other foreign material; nor did I fine any trace of a stopping either among the ashes or in the teeth of the unburnt bodies, although I have heard of the assertion that gold or other stoppings were to be found in Roman teeth. Several eminent antiquaries whom I have consulted have assured me they had never noticed any traces of either mechanical or surgical operations upon the teeth in Roman skulls. I may, however, here

* I was informed by Dr. Birch, of the British Museum, of the existence of a curious passage in the old Roman laws of the twelve tables, B.C. 450. These laws were inscribed upon bronze tablets, and perished in the destruction of Rome by the Goths. I was aided by a kind friend in my search, who discovered the passage in one of the fragmentary quotations by Cicero (De Legibus, ii. 24). The passage stands thus, in old law Latin, "Neve aurum addito. Quoi auro dentes vincti escunt ast im cum illo sepelire urereve se fraude esto." In classical Latin the passage would read thus: "Neve aurum addito. Qui auro dentes vincti erunt, at eum cum illo sepelire urereve sine fraude esto." And may be thus translated: "Neither add any gold (to a corpse), but if any one shall have teeth bound with gold, it shall be no offense to bury or burn him with it." Was this a case of tying in artifical teeth with gold wire, or an anticipation of the modern advertisement of "Loose teeth fastened?"

observe, that the molar teeth had frequently been removed by operation, as the rounded form of the jaw-bone presented a total contrast in these cases to those in which they had been lost through absorption of the sockets arising from diseased stumps.*

Before I leave this part of my subject, I will state the result of my investigations (with a similar object) in reference to the skulls of ancient Egyptians. Although so many Egyptian mummies are to be found in our various museums, the proportion is small in which they have been sufficiently unrolled to admit of a thorough examination of the teeth. I have, however, been fortunate enough to be able to examine the skulls of thirty-six mummies, eleven of which exhibit signs of disease on the approximal surfaces of the teeth, and in two cases the upper incisors were involved. I also noticed a case of irregularity in the upper jaw, in which a supernumerary tooth appeared between

* In order to ascertain, if possible, whether any record existed of the discovery of relics of the art of these ancient dentists, I have thoroughly examined many extensive works on Roman antiquities, including Sir W. Gell's "Pompeii," Bosio's "Roma Sotterranea," Beckman's "History of Inventions," and several modern works. I have also carefully looked over the remains of Roman art in several collections, and although I found probes and needles, strigils for scrapping the skin in the bath, instruments for trimming and cleaning the nails, tweezers and mirrors of bronze and other metals, pins and combs of box-wood and bone, with other appliances of the toilet in abundance; fibulæ, bracelets, finger-rings, and ear-rings in gold, and other articles of adornment, I found not a trace of an artificial tooth or its setting, or any instrument that I could suppose was intended for operating on the teeth. There are, however, existing numerous mementoes of another class of practitioners, who must have carried on an extensive business much after the plan of our modern proprietory medicine-dealers; and it is a curious fact that diseases of the eye are the only maladies referred to in these relics, which are known to antiquarians as oculists' stamps. Although not directly connected with my paper, I may observe that numerous examples of them have been found, not only in Italy, but in Germany, France, and Britain; they are usually cut out of some soft stone, such as steatite, with the letters in intaglio, or sunken, so that an impression on the cover of the medicine was made, probably in some plastic material. From the testimony of Pliny, and other ancient writers, we know that diseases of the eye were very prevalent, the Greek medical authors enumerating more than 200 varieties. The remedies, or "collyria," as they were called, were composed of a great variety of ingredients, which were often indicated upon the inscription, with the name of the practitioner. Antiquaries have supposed that the prevalence of ophthalmic diseases, not only in Italy, but throughout the western provinces, was due to some circumstances connected with the diet or manners of the ancients. I would, however, suggest that as no record exists, as far as I am aware, of the use of spectacles until the fifteenth century—at the very earliest—we may reasonably conclude that the gradual flattening of the crystalline lens with advancing age, will account for a very large proportion of these maladies.

the central incisors, and both laterals were absent. In a large proportion of these cases the teeth had suffered greatly from attrition—a condition which confirms Blumenbach's remarks, although not his deduction; and on conversing with Mr. Bonomi, the eminent Egyptian scholar, who lived many years among the tombs, he assured me that he had examined the teeth in at least 500 skulls, and found that truncated condition of the teeth very prevalent.

We learn from Herodotus, that a special class of practitioners gave attention to the teeth, and I have found repeated instances in which teeth had evidently been removed by an operation; but after a diligent perusal of the works of Belzoni, Sir Gardner Wilkinson, Dr. Pettigrew, and other writers, I can find no authority for the assertion that the Egyptians made artificial teeth of ivory or box-wood, or that they practiced the art of stopping, and Mr. Bonomi assures me that he has never seen either an artificial tooth or a stopping in a single instance, although he often noticed cases of the absence and decay of teeth. The Egyptians were in the habit of gilding the eyelids, lips, and nose of their mummies, and a curious practice also occasionally prevailed of laying a thin plate of pure gold upon the tongue, inscribed with hieroglyphic characters, and these practices may have given rise to the mistake. An Arab removed and sold three of these, which weighed together 9 mithkals, which I have calculated to give 6 dwts. 16 grs. as the average weight of each plate. This is the only gold object I can discover to have been found in the mouth of a mummy.

Although I have failed in my researches to find any trace of the practice of dental surgery, excepting the removal of teeth—or any example of dental mechanics, among the ancient Egyptians and Romans—it is something attained, perhaps, to be able to infer that the question of their alleged practice of gold-stopping is, in all probability, settled in the negative. Had these operations been often performed, it is fair to suppose that some proof would have been found among the many skulls I have examined in this inquiry.

Mr. Bonomi explains the great amount of attrition of the teeth as the result of the inevitable mixture of sand with the food of the Egyptians, their mode of grinding the corn being so very slow and imperfect. He had himself seen in Upper Egypt the natives bruising their corn and reducing it to a very coarse flour, by the use of the primitive method of triturating it on a granite slab with a rude pestle, a mode depicted on the ancient Egyptian monuments. The food of the higher classes of the Egyptians appears to have been luxurious; they had abundance of corn and pulse, and a great variety of vegetables, besides the flesh of the ox, antelope, ibex, and other animals, with many kinds of fish. I found a great difference in the condition of the teeth in various groups of skulls; probably owing to different habits of the particular district. In one collection of ten skulls, three cases of disease occurred, and these of slight character. In another collection of nine skulls, I found approximal caries, often very extensive, in no less than eight; the upper incisors being diseased in two cases.

I cannot pursue my subject further this evening. I hope, however, at our next meeting, to give due attention to the condition of the teeth in Anglo-Saxon times, and also to adduce, from a comparison of the teeth of existing races with those of ancient peoples, abundant proof that dental disease is not exclusively the accompaniment of a high degree of civilization, although the evidence I have brought before you up to the present point appears to favor this idea. I shall be able on that occasion to bring examples of the liability to dental caries among nations in the rudest state of society at the present day; and to show that, with suitable diet and attention to the general laws of health, a sound condition of the teeth is not always incompatible with intellectual advancement.—*To be Continued.*—*Trans. Odonto. Society, Great Britain.*

